# Supplementary material for: The effect of adjuvant therapy for patients with intrahepatic cholangiocarcinoma after surgical resection: A systematic review and meta-analysis
Source: PLoS One. 2020 Feb 21;15(2):e0229292. doi: 10.1371/journal.pone.0229292 (PMC7034847; doi:10.1371/journal.pone.0229292)
Supplement: S2 Table — (DOCX) [file pone.0229292.s002.docx]

**Search strategy**

| **Database** | **Strategy** |
| --- | --- |
| Pubmed | #1 “intrahepatic cholangiocarcinoma”[MeSH Terms] OR “intrahepatic cholangiocarcinoma”[All Fields] |
|  | #2 “adjuvant therapy”[MeSH Terms] OR “adjuvant therapy”[All Fields] OR “adjuvant treatment”[MeSH Terms] OR “adjuvant treatment”[All Fields] |
|  | #3 “transarterial chemoembolization”[MeSH Terms] OR “transarterial chemoembolization”[All Fields] |
|  | #4 “chemotherapy”[MeSH Terms] OR “chemotherapy”[All Fields] |
|  | #5 “radiotherapy”[MeSH Terms] OR “radiotherapy”[All Fields] |
|  | #6 “chemoradiotherapy”[MeSH Terms] OR “chemoradiotherapy”[All Fields] |
|  | #7 #1 and #2 |
|  | #8 #1 and #3 |
|  | #9 #1 and #4 |
|  | #10 #1 and #5 |
|  | #11 #1 and #6 |
