# Supplementary material for: The effect of adjuvant therapy for patients with intrahepatic cholangiocarcinoma after surgical resection: A systematic review and meta-analysis
Source: PLoS One. 2020 Feb 21;15(2):e0229292. doi: 10.1371/journal.pone.0229292 (PMC7034847; doi:10.1371/journal.pone.0229292)
Supplement: Attachment — Submitted filename: Response to Reviewers.docx [file pone.0229292.s003.docx]

Dear editor,

Firstly, we would like to thank the reviewers and the editor for the positive and constructive comments and suggestions. We have substantially revised our manuscript after reading the comments, and the revision was marked in red. In addition, the revision was re-edited literally, and the part of the method, especially the statistics, was checked repeatedly.

Point by point responses to reviewers:

**Journal Requirements:**

When submitting your revision, we need you to address these additional requirements:

1. Please ensure that your manuscript meets PLOS ONE's style requirements, including those for file naming. The PLOS ONE style templates can be found at http://www.plosone.org/attachments/PLOSOne_formatting_sample_main_body.pdf and [http://www.plosone.org/attachments/PLOSOne_formatting_sample_title_authors_affiliations.pdf](http://www.plosone.org/attachments/PLOSOne_formatting_sample_title_authors_affiliations.pdf)

Response: Thank you for your kindly reminder. We are sure that our manuscript is well meet the requirements of PLOS ONE.

2. At this time, we ask that you please provide the full search strategy and search terms for at least one database in the Supplementary information.

Response: Thank you for your professional comment. Full search strategy and search terms for PubMed was uploaded as the Supplementary file. However, manually searching was repeated conducted through the references of the included studies, reviews, letters and comments.

3. Thank you for stating the following beneath the Acknowledgments Section of your manuscript:

"This study was supported by Startup Fund for scientific research, Fujian Medical University (Grant number: 2018QH1195)."

We note that you have provided funding information that is not currently declared in your Funding Statement. However, funding information should not appear in the Acknowledgments section or other areas of your manuscript. We will only publish funding information present in the Funding Statement section of the online submission form.

Please remove any funding-related text from the manuscript and let us know how you would like to update your Funding Statement. Currently, your Funding Statement reads as follows:

"Not application"

a. Please provide an amended Funding Statement that declares *all* the funding or sources of support received during this specific study (whether external or internal to your organization) as detailed online in our guide for authors at [http://journals.plos.org/plosone/s/submit-now](http://journals.plos.org/plosone/s/submit-now)
b. Please state what role the funders took in the study.  If any authors received a salary from any of your funders, please state which authors and which funder. If the funders had no role, please state: "The funders had no role in study design, data collection and analysis, decision to publish, or preparation of the manuscript."
c. Please include your amended statements within your cover letter; we will change the online submission form on your behalf.

Response: Thank you for your kindly reminder. Lei Wang is the grantee of the funding, but "The funders had no role in study design, data collection and analysis, decision to publish, or preparation of the manuscript." We removed funding-related text from the manuscript and update our Funding Statement as you suggested.

4. PLOS requires an ORCID iD for the corresponding author in Editorial Manager on papers submitted after December 6th, 2016. Please ensure that you have an ORCID iD and that it is validated in Editorial Manager. To do this, go to ‘Update my Information’ (in the upper left-hand corner of the main menu), and click on the Fetch/Validate link next to the ORCID field. This will take you to the ORCID site and allow you to create a new iD or authenticate a pre-existing iD in Editorial Manager. Please see the following video for instructions on linking an ORCID iD to your Editorial Manager account: <https://www.youtube.com/watch?v=_xcclfuvtxQ>

Response: Thank you for your kindly reminder. We have added all the authors’s ORCID and updated our information.

**Reviewer #1:** I read the manuscript Ke et al with great interest. The authors tried to answer the question whether adjuvant therapy (in different forms) for intrahepatic CCA would be useful or not using the meta-analysis of the published paper.With the large cohort of the meta-analysis and included study it looks like the statistical power seems adequate. However, the authors included studies which span over 30 years.
I have several comments;
1- Due to 30 years of span (1990-2019), as also can be seen by Table 2, some chemotherapy agents were different. Therefore, the response to chemotherapy could be different. This should be acknowledged in the discussion as the most limiting factor for chemotherapy and chemoradipotherapy. This long time span eventually impacts on the surgical techniques as well. The power of meta-analysis is the large cohort but different surgical techniques, (improved techniques most recently with improved diagnostics) change the overall survival of CCA patients. This should be also acknowledged as an important limitation.
Response: Thank you for your professional comment. Firstly, we admitted that the span of the included researches was a little longer. Considering that most of the included studies were after 2010, we conducted a subgroup analysis stratified by the publication year before and after 2010 as you suggested, and results showed that the results of two subgroup analysis were coincident with the whole, which indicated that the conclusion was robust to some extent. Secondly, as you indicated that “some chemotherapy agents were different “, and the relevant subgroup analysis was published previously, which was depicted in the “Discussion”. And, the emphasis and innovation were the subgroup analysis stratified by different adjuvant strategies including chemotherapy, TACE, radiotherapy, and chemoradiotherapy. Thirdly, as you pointed out that “different surgical techniques, (improved techniques most recently with improved diagnostics) change the overall survival of CCA patients”, but relevant subgroup analysis such as laparoscopic vs. open hepatectomy, major vs. minor hepatectomy, hepatectomy with or without lymphadenectomy were unable to conduct due to most of the data was unavailable. However, these limitations were emphasized in the “Discussion” as you suggested.
2- page 3, line 70 - Varies should be Various

Response: Thank you for kindly reminder, and we corrected it in the revised manuscript.
3- page 4 line 77, OMIT, "badly".
Response: Thank you for kindly reminder, and we corrected it in the revised manuscript.
Again, the authors should add limitations to the study and soften the conclusion based on these limitations.
Response: Thank you for kindly reminder, and we had softened our conclusion in the revised manuscript.

**Reviewer #2:** This manuscript by Qiao Ke et al. is a meta-analysis evaluating the effect of adjuvant therapy (AT) for patients with intrahepatic cholangiocarcinoma (ICC) after resection. Resection is still the only potentially curative treatment for patients with intrahepatic cholangiocarcinoma (ICC), but the prognosis remains far from satisfactory. The role of conventional therapies (transarterial chemoembolization, chemotherapy and radiotherapy) has yet to be fully defined, particularly in the adjuvant and second-line settings. This is the first systematic review evaluating the clinical value of AT in the treatment of ICC, including 22 studies with 10181 patients. Results showed that some patients could be benefited from AT in a whole. However, not all AT strategies would bring benefit to patients with ICC, and the benefit of adjuvant radiotherapy needs to be further validated. Identifying the selected patients and choosing the appropriate AT strategy is a major challenge for Clinics. The manuscript is well written and easy to follow. The topic of this manuscript is of great interest. The Reviewer has just some issues to address.
MAJOR
1. The major weaknesses of this study are well addressed by the Authors in the Discussion chapter: the studies are retrospective, indicating an obvious selection and recalling bias; most of the studies are multi-centers or based on the database mainly due to the rare incidence of ICC, which meant that procedure of surgical resection and AT were different and bias was hard to avoid; most of the cofounding factors such as radical resection and LNM are hard to be resorted in the original studies, which would weaken the conclusion; RFS is evaluated in only six of the 22 included studies, which is insufficient to evaluate the effect of AT on recurrence; publication bias is found in this meta-analysis.

Response: Thank you for your professional comments, and these limitations above were hardly avoided, and would weaken the conclusion of this study. Hence, we had softened the conclusion in the revised manuscript.
2. Since a number of pathway-targeted therapies, as well as modulation of the immune environment, hold promise for patients with intrahepatic cholangiocarcinoma, the Authors should add this topic in the discussion.

Response: Thank you for your professional comment. Considering the great advances in the pathway-targeted therapies and immunotherapies, prognosis of patients with intrahepatic cholangiocarcinoma following resection would be improved. Hence, we checked the latest reports on this topic, and added the progress in the revised “Discussion”.

We are looking forward to receive your letter, and please contact me without any hesitation if you have any question.

Best Regards,

Yours sincerely,

Qiao Ke

Corresponding to Lei Wang, E-mail: wangleiy001@126.com
